# CircRNA ITCH: Insight Into Its Role and Clinical Application Prospect in Tumor and Non-Tumor Diseases

**DOI:** 10.3389/fgene.2022.927541

**Published:** 2022-07-15

**Authors:** Tong Liu, Tao Huang, Mei Shang, Gang Han

**Affiliations:** Department of Gastrointestinal Nutrition and Hernia Surgery, The Second Hospital of Jilin University, Changchun, China

**Keywords:** circ-ITCH, malignant tumor, non-tumor diseases, mechanism, biomarkers

## Abstract

CircRNA E3 ubiquitin protein ligase (ITCH) (circRNA ITCH, circ-ITCH), a stable closed-loop RNA derived from the 20q11.22 region of chromosome 20, is a new circRNA discovered in the cytoplasm in recent decades. Studies have shown that it does not encode proteins, but regulates proteins expression at different levels. It is down-regulated in tumor diseases and is involved in a number of biological activities, including inhibiting cell proliferation, migration, invasion, and promoting apoptosis. It can also alter disease progression in non-tumor disease by affecting the cell cycle, inflammatory response, and critical proteins. Circ-ITCH also holds a lot of promise in terms of tumor and non-tumor clinical diagnosis, prognosis, and targeted therapy. As a result, in order to aid clinical research in the hunt for a new strategy for diagnosing and treating human diseases, this study describes the mechanism of circ-ITCH as well as its clinical implications.

## 1 Introduction

In brief, diseases were categorized into two groups: tumors and non-tumor disorders. Malignant tumors, for example, are a type of incurable polygenic disease that has claimed the lives of millions of individuals worldwide. According to GLOBOCAN 2020, there were 19.3 million new malignant tumor cases and 9.9 million deaths globally in 2020 (Sung et al., 2021). Although surgical resection and advanced therapeutic interventions have improved the 5-year survival rate in patients with early-stage GC, the prognosis for late-stage GC patients remains poor due to uncontrolled tumor cell growth and migration (Lasithiotakis et al., 2014). Non-tumor disorders, such as degenerative, metabolic, congenital, and inflammatory pathologies, make up the great bulk of all pathologies, aside from tumors. As a result, finding effective diagnostic biomarkers and treatment targets is crucial for disease fundamental research.

Benefiting from high-throughput sequencing, researchers can take a nuanced and complete picture of the transcriptome and genome of a species. RNA sequencing (RNA-seq) technology has become one of the important means of transcriptomic studies of high-throughput sequencing, which can discover all RNAs that a particular cell can transcribe in a certain functional state, mainly including mRNAs and non-coding RNAs, while avoiding detection using standard molecular techniques. CircRNAs, which were previously thought to be misspliced products, have lately been shown to have a range of biological regulatory activities owing to the development of RNA-seq (Memczak et al., 2013). Most circular RNAs (circRNAs) are mainly composed of one or more exons encoding known proteins. The 3' and 5' terminals of circRNAs are covalently bonded to form a closed-loop structure, unlike typical linear RNAs. It has no free terminal and is unaffected by RNA exonuclease, resulting in a more stable and difficult-to-degrade copy. CircRNAs is primarily involved in the following four processes: 1) sponging microRNAs (miRNAs) or long noncoding RNAs (lncRNAs) as a competing endogenous RNA (ceRNA); 2) binding RNA binding proteins (RBPs); 3) interfering with gene transcription and splicing regulation; and 4) translating protein/polypeptide (Kristensen et al., 2019). Maass et al. (2017) identified 71 differentially expressed circRNAs in 20 human clinical samples including tissues, blood, proving that circRNAs can express stably *in vitro* and are associated with multiple diseases, which has good potential for biomarker development.

**FIGURE 1:**
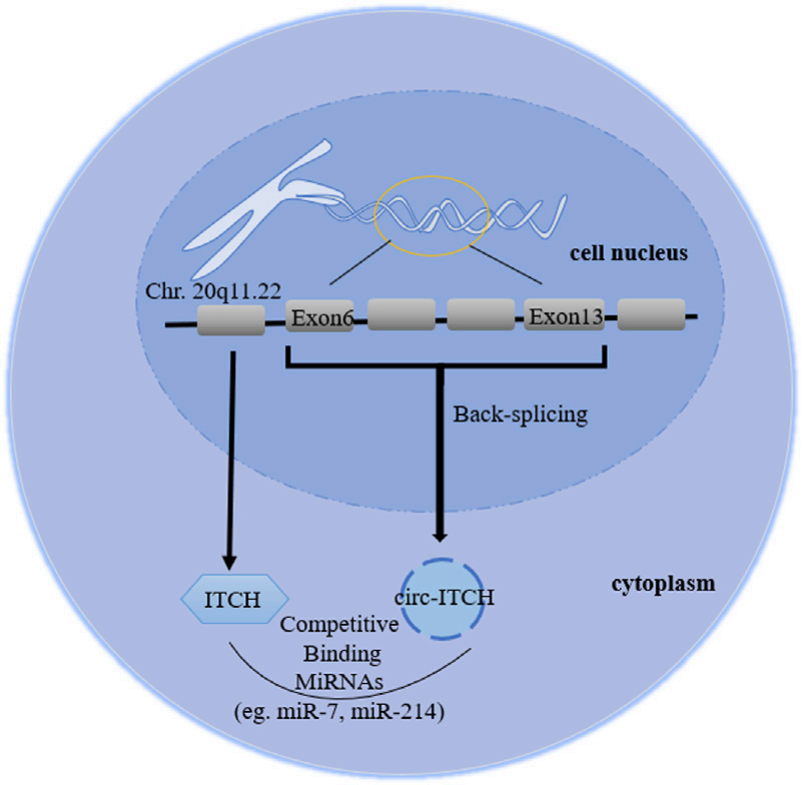
Biogenesis diagram of circ-ITCH. The ID number of circ-ITCH is hsa_circ_0001141, whose gene is located in chr 20q11.22, derived from exon 6–13 of ITCH coding gene, formed by back-splicing, and its mature sequence is 873bp.

As a member of the E3 ubiquitin protein ligase (ITCH) HECT family, ITCH can ubiquitinate phosphorylated disheveled-2 (Dvl2), promoting its degradation (Bernassola et al., 2008). Phosphorylated Dvl2 is the upstream target of activating β-catenin in the canonical Wnt pathway, therefore ITCH can block the canonical Wnt pathway, regulating cell cycle (Li et al., 2015). In addition, ITCH can regulate immune responses, epidermal keratinocyte differentiation and receptor trafficking/signaling (Melino et al., 2008). Previous studies have shown that in ITCH^−/−^ mice, some signal proteins (such as Jun family members and Notch) are abnormally accumulated, seriously affecting the autoimmune phenotype (Parravicini et al., 2008). Jun and Notch are also transcription factors that control the maintenance of epidermal stem cells and the regulation of keratinocytes. The degradation of these proteins mediated by ITCH may play a regulatory role in skin biology (Candi et al., 2005), indicating that it has certain potential in radiotherapy protection. Moreover, Sundvall et al. (2008) emphasized the role of pruritus in regulating the endocytosis and protein stability of erbb-4, a receptor belonging to the epidermal growth factor receptor (EGFR)/ErbB family. Therefore, ITCH involves a variety of physiological and pathological regulation through different mechanisms, including regulating Wnt, Jun, Notch, MAPK signaling, immune cells differentiation and EGFR family. CircRNA E3 ubiquitin protein ligase (circRNA ITCH, hereinafter referred to as circ-ITCH), a stable closed-loop RNA with no protein coding ability and derived from the 20q11.22 region of chromosome 20, is a new circRNA discovered in recent decades (Bernassola et al., 2008; Memczak et al., 2013). The initial study reported that circ-ITCH came from exon 6–13 of E3 ubiquitin protein ligase (ITCH) encoding gene (Li et al., 2015), but then it became exon 7–14 in related studies without any explanation (Han et al., 2020), which is a point that needs to be clarified in subsequent experiments. Combining the database (Circular RNA Interactome and circBase) and related literature search, we considered it derived from exons 6–13 (Figure 1), while 7–14 was a writing error in correlative papers. Recently, studies have revealed that circ-ITCH is down-regulated in multiple tumor tissues, and regulates cell proliferation, migration, invasion and apoptosis of malignant tumor, indicating it might be an important tumor suppressor (Li Y. et al., 2020; Ghafouri-Fard et al., 2021; Su et al., 2022). Additionally, the down-regulation can also be seen in the peripheral blood and exosomes of patients. Its low expression has some diagnostic relevance and is linked to negative clinical outcomes (such as tumor sizes, lymph node metastasis and distant metastasis). Furthermore, circ-ITCH plays an essential function in non-tumor illnesses. Understanding its function and mechanism could help clinical researchers discover novel strategies to diagnose and treat a variety of diseases earlier.

## 2 Regulation Mechanism and Role of Circ-ITCH in Tumor Diseases

MiRNA, as a part of the ceRNA network, causes polyadenylation by binding with target sites in the 3'-UTR of mRNA, lowering mRNA stability and interfering with translation to adversely control gene expression (Nawaz et al., 2016). CircRNAs may have biological impacts on tumors by sponging target miRNAs and limiting their function, according to previous studies (Li Y. et al., 2020; Ghafouri-Fard et al., 2021; Su et al., 2022). There is no exception for circ-ITCH. MiRNAs sponge locations of circ-ITCH downstream in malignant tumors now being researched, including miR-7, miR-10, miR-17, miR-20, miR-22, miR-93, miR-106, miR-145, miR-199, miR-214, miR-216, miR-224, miR-421, miR-524, and miR-615 (Huang et al., 2015; Li et al., 2015; Luo et al., 2018b; Hu et al., 2018; Wang et al., 2018; Yang et al., 2018; Lin et al., 2020; Liu et al., 2020; Wang et al., 2021). The specific regulation mechanism of circ-ITCH in a range of malignant tumors is also variable due to sponging distinct miRNAs (Table 1).

**TABLE 1:** Anti-tumor mechanism of circ-ITCH in a variety of malignant tumors.

Tumor types	ceRNA	Effect	Ref
OC	miR-106a, miR-145 and lncRNA HULC	proliferation↓; migration↓; invasion↓; apoptosis↑; glycolysis↓	Lin et al., 2020; Hu et al., 2018; Yan et al., 2020
ESCC	miR-7, miR-17 and miR-214	proliferation↓	Li et al. (2015)
CRC	miR-7, miR-20a and miR-214	proliferation↓	Huang et al. (2015)
BCa	miR-17, miR-224	apoptosis↑; proliferation↓; invasion↓; migration↓	Yang et al. (2018)
PTC	miR-22-3p	apoptosis↑; proliferation↓; invasion↓	Wang et al. (2018)
EOC	miR-10a-α	apoptosis↑; proliferation↓	Luo et al. (2018b)
GC	miR-199a-5p, miR-17	proliferation↓; migration↓; invasion↓; EMT↓	Peng et al., 2020; Wang et al., 2021
MM	miR-615-3p	apoptosis↑; proliferation↓; BTZ chemosensitivity↑	Liu et al. (2020)
TNBC	miR-17, miR-214	proliferation↓; migration↓; invasion↓	Wang et al. (2019a)
HCC	miR-7, miR-214, miR-421 and miR-224-5p, miR-184	apoptosis↑; proliferation↓; migration↓; invasion↓	Yang et al., 2020; Wu et al., 2020; Zhao et al., 2021; Guo et al., 2022
LC	miR-7, miR-214	proliferation↓	Wan et al. (2016)
Glioma	miR-106a-5p	proliferation↓; migration↓; invasion↓; apoptosis↑	Chen et al. (2021)
PCa	miR-17-5p, miR-197	proliferation↓; migration↓; invasion↓; apoptosis↑	Wang et al., 2019b; Yuan et al., 2019
Melanoma	miR-660	proliferation↓; migration↓	Zhang et al. (2022)
OS	miR-22, miR-524	proliferation↓; migration↓; invasion↓; apoptosis↑	Ren et al., 2019; Li et al. (2020a)
ccRCC	miR-106b-5p	proliferation↓; migration↓; invasion↓	Gao et al. (2021)
OSCC	miR-421	apoptosis↑; proliferation↓	Hao et al. (2020)
CC	miR-93-5p	proliferation↓; migration↓; invasion↓	Li et al. (2020b)
NPC	miR-214	proliferation↓; migration↓; invasion↓	Wang et al. (2022)

### 2.1 Regulating Canonical Wnt Signaling Pathway

The Wnt/β-catenin signaling pathway is highly conserved and important for cell motility, invasion, polarity formation, organogenesis, and cell stemness maintenance (Wei et al., 2012). The Wnt/β-catenin signaling pathway has two pathways: the canonical pathway and the non-canonical pathway, with the conventional pathway being the more common (Wei et al., 2012; Muralidhar et al., 2019). Canonical signal pathways consist of the Wnt protein, Wnt receptor [frizzled family protein (FZD) and low density lipoprotein receptor associated protein-5/6 (LRP-5/6)], Dvl2, β-catenin protein and et al. (Muralidhar et al., 2019) Wnt protein interacts with the FZD receptor on the cell membrane surface in an autocrine or paracrine manner, then recruits LRP-5/6 to create a complex that activates intracellular Dvl2 protein via a phosphorylation cascade. Through its PDZ domain (one of Dvl2's domains), phosphorylated Dvl2 favorably regulates β-catenin protein, facilitating its entry into the nucleus as a transcriptional regulator and activating the expression of downstream target genes including cyclinD1 and c-Myc (Muralidhar et al., 2019).

Through targeting certain miRNAs, Circ-ITCH inhibits the function of matching miRNAs, particularly miRNAs that inhibit linear ITCH. Specifically, researchers showed that reporter gene assays in the presence of circ-ITCH demonstrated that the inhibitory effects of different miRNAs (including miR-7, miR-17, miR-20a, miR-22-3p, and miR-214) were dampened by the co-expression of circ-ITCH, consistent with the “sponge” hypothesis (Huang et al., 2015; Li et al., 2015; Wang et al., 2018). Among these miRNAs, the miR-7 and miR-214 are most important because they can share the binding sites with the 3′-UTR of circ-ITCH and its parental gene ITCH (Verduci et al., 2021). It is worth mentioning that, as one of the most conservative and oldest miRNAs, miR-7 plays different roles in different cancers and participates in many signal pathways involving differentiation, proliferation regulation, apoptosis and migration. In most tumors, its expression is down-regulated because its main activity is to inhibit tumor by inhibiting cell proliferation and survival. However, in lung cancer and oral cancer, its expression is up-regulated as a carcinogen, which is consistent with the research on circ-ITCH (Kora´c et al., 2021). Since circ-ITCH and ITCH share the 3'-UTR of miR-7, they will produce competitive inhibition. When circ-ITCH is up-regulated, the remaining ITCH content *in vivo* will be up-regulated (Verduci et al., 2021). ITCH can identify and ubiquitinate a range of proteins, the most important of which is phosphorylated Dvl2 (Wei et al., 2012). It is well known that Dvl family proteins are mostly made up of Dvl1-3, with Dvl2 serving as a key scaffold in the canonical Wnt pathway, connecting upstream Wnt protein with downstream β-catenin protein (Wei et al., 2012). As a result, below is the whole regulator mechanism: via sponging miR-7, miR-17, miR-20a, miR-22-3p, and miR-214, circ-ITCH increases ITCH levels. While phosphorylated Dvl2 labeled by ITCH ubiquitin promoted its degradation and inhibited the canonical Wnt pathway. A summary of recent studies has found that in esophageal squamous cell carcinoma (ESCC), colorectal cancer (CRC), lung cancer (LC), three negative breast cancer (TNBC), prostate cancer (PCa), hepatocellular carcinoma (HCC) and gastric cancer (GC), circ-ITCH could up-regulate the expression of linear ITCH via sponging miR-7, miR-17 and miR-20a, thereby inhibiting the canonical Wnt pathway and further suppressing the activation of c-Myc and cyclinD1 (Wan et al., 2016; Wang S. et al., 2019; Li S. et al., 2020; Peng and Wang, 2020; Yang et al., 2020). As widely reported, c-Myc is an oncogene. Its aberrant activation frequently results in unrestricted cell proliferation and immortalization, promoting cell malignancy and tumorigenesis (Glöckner et al., 2002). CyclinD1 (also known as G1/S-specific cyclin D1), on the other hand, regulates the cell cycle and promotes cell proliferation, and is up-regulated in a number of malignancies (Glöckner et al., 2002). In addition, Wang et al. (2018) also confirmed that circ-ITCH can sponge miR-22-3p and increase the expression of CBL in papillary thyroid cancer (PTC), inhibiting cell proliferation and invasion, increasing apoptosis, and repressing PTC progression. CBL is also a member of the E3 ubiquitin ligase family, which can ubiquitinate as well as label β-catenin to promote its destruction and so block the Wnt pathway (Shashar et al., 2016). In most tumors, the glucose transporter 1 (GLUT1) gene is overexpressed. By mediating glucose via the plasma membrane and increasing glucose absorption, it plays a vital function in the early stages of intracellular glucose metabolism and promotes tumor growth (Koch et al., 2015). ITCH can down-regulate the expression of GLUT1 in melanoma, reducing glucose uptake and tumor cell growth, according to Lin et al. (2021), but whether this regulation also involves the Wnt pathway needs to be confirmed in further experiments.

### 2.2 Regulating PI3K/Akt Signaling Pathways and MEK/Erk Cascade

In the progression of many malignancies, activation of the PI3K/Akt pathway and MEK/Erk cascade have been confirmed. Following activation, PI3K activates the Akt protein, which subsequently enters the nucleus and regulates cell proliferation, invasion, migration, metabolic reprogramming, autophagy, and aging, potentially causing malignant tumors (Hermida et al., 2017). The MEK/Erk cascade interacts closely with the PI3K/Akt cascade and is involved in tumor development. After activating signaling pathways, many phosphorylated Erk substrates have been shown to contribute to cell proliferation and invasion (Burotto et al., 2014). Phosphatase and tensin homolog deleted on chromosome ten (PTEN) is a miR-7, miR-22, and miR-224 target that inhibits the PI3K/Akt cascade (Sadri Nahand et al., 2021). P21 is the downstream target of the PI3K/Akt cascade. To promote cell proliferation, activated Akt can phosphorylate p21, blocking its cell cycle arrest function (Cheng et al., 2019). Circ-ITCH sponges these miRNAs to up-regulate PTEN expression in bladder cancer (BCa) and OS, blocking the PI3K/Akt cascade, and up-regulating p21 protein to prevent tumor cell proliferation, migration, invasion and promote apoptosis (Yang et al., 2018; Ren et al., 2019). A recent vitro experimental investigation found that circ-ITCH might further up-regulate PTEN in nasopharyngeal cancer (NPC) via sponging miR-214 (Wang et al., 2022), indicating that it can prevent NPC progression by blocking the PI3K/Akt pathway. Ras p21 protein activator 1 (RASA1) is a regulator of Ras-GDP and GTP, which promotes apoptosis and inhibits angiogenesis, cell proliferation by inhibiting Ras/Raf/MEK/Erk signals cascade (Zhang et al., 2020). RASA1 has been shown to be low expressed in a number of tumors, and miR-14 has been identified to mute it (Zhang et al., 2020). Additionally, Hu et al. (2018) found that in ovarian cancer (OC), circ-ITCH up-regulated RASA1 by sponging miR-145, blocking the PI3K/Akt pathway and MEK/Erk cascade, therefore decreasing tumor cell malignancy. Yan et al. (2020) found a negative connection between circ-ITCH expression and lncRNA HULC expression in OC. Previous research has demonstrated that through down-regulating the miR-125a-3p level, lncRNA HULC can activate the PI3K/Akt/mTOR pathway, promoting the proliferation, migration, and invasion of OC cells (Chu et al., 2019). Therefore, circ-ITCH might compete with lncRNA HULC for miR-125a-3p binding, blocking the PI3K/Akt/mTOR pathway and so acting as an anti-tumor agent. However, the hypothesis needs to be confirmed by further experiments. Published reports by two independent groups of researchers in 2019 and 2021 suggested that circ-ITCH expression decreased in patients’ OS sample tissue, and that circ-ITCH hindered the proliferation, migration, and invasion of OS cells via sponging miR-22 and miR-524 (Ren et al., 2019; Zhou W. et al., 2021). But interestingly, in 2020, Li et al. (Li H. et al., 2020) discovered that the expression of circ-ITCH was up-regulated in U2OS and SJSA-1 cell lines, and enhanced the expression of epidermal growth factor receptor (EGFR) by reducing the level of tumor suppressor miR-7 in OS. Then, when EGFR is overexpressed, it activates the PI3K/Akt and MEK/Erk pathways, promoting OS development. This finding contradicts the findings of two previous investigations. However, there is a flaw in the experiment: it did not verify the degree of circ-ITCH expression in the OS tissue sample. It's possible that this is due to the heterogeneity of OS cell lines or the complexity of studying the regulatory network. Consequently, in the research of circ-ITCH in OS, more parameters should be explored.

### 2.3 Regulating Cell Cycle-Related Proteins

Programed cell death receptor 4 (PDCD4) is described as a tumor suppressor, which is often down-regulated in tumors, promoting tumor cell apoptosis and inhibiting its proliferation, invasion and metastasis (Yang et al., 2021). MiR-106b-5p and miR-421 are common upstream targeting miRNAs of PDCD4 and can inhibit its expression (Wang Y. et al., 2019; Yang et al., 2021). Circ-ITCH specifically targets miR-106b-5p and miR-421 in clear cell renal cell carcinoma (ccRCC) and oral squamous cell carcinoma (OSCC) to up-regulate PDCD4 expression and prevent tumor progression, respectively (Hao et al., 2020; Gao et al., 2021). RAS association domain family member 6 (RASSF6) inhibits cell growth and promotes apoptosis in a variety of tumors by interrupting the cell cycle (van der Weyden and Adams, 2007). In OS, circ-ITCH sponges miR-524 to up-regulate RASSF6, inducing OS cell death and limiting its proliferation, according to Zhou et al. (Zhou W. et al., 2021). SAM and SH3 domain containing protein 1 (SASH1) is a tumor-suppressive protein that can regulate cell apoptosis and proliferation (Burgess et al., 2020). Circ-ITCH can suppress glioma growth and invasion by up-regulating SASH1 by targeting miR-106a-5p (Chen et al., 2021). Cytoplasmic polyadenylation element binding protein 3 (CPEB3), a RNA binding protein, plays a tumor-suppressive role though regulating the expression of malignant transformation-related genes through post-transcriptional control (Pichon et al., 2012). In HCC, circ-ITCH binds to miR-421 to prevent CPEB3 down-regulation and tumor growth (Zhao et al., 2021). MafF belonging to the Maf family, a basic leucine zipper (bZIP) transcription factor, has been found to have anti-tumor properties in HCC (Tsuchiya and Oura, 2018). By modulating the miR-224-5p/MAFF axis, Circ-ITCH can also decrease cell growth and increase apoptosis (Wu et al., 2020). Li et al. (2020b) found that circ-ITCH sponges miR-93-5p to up-regulate forkhead box K2 (FoxK2) and block tumor growth in cervical cancer (CC). Simultaneously, FoxK2 expression was dramatically reduced in CC tissues, and miR-93-5p mimic transfection further decreased FoxK2 expression in CC cell lines. FoxK2 deletion improved the capacity of cells transfected with miR-93-5p mimic to invade. Homeobox B13 (HOXB13), according to earlier research, inhibits the cell cycle by promoting the ubiquitination and degradation of cyclinD1 in a variety of malignancies (Hamid et al., 2014). Circ-ITCH restrains PCa cell proliferation, invasion, and migration via sponging miR-17-5p and boosting HOXB13 up-regulation, as well as promoting apoptosis (Wang X. et al., 2019). Furthermore, via targeting miR-197, circ-ITCH can attenuate PCa cell proliferation and increase apoptosis, however the underlying mechanism is uncertain (Yuan et al., 2019). In melanoma, circ-ITCH suppresses cell proliferation and metastasis through sponging miR-660, a previously reported tumor-promoting miRNA, further up-regulating transcription factor cellular promoter 2 (TFCP2) (Zhang et al., 2022). TFCP2, as a cell cycle regulating molecule, mainly plays a tumor suppressor role in melanoma. Its role is mainly to positively regulate the DAPK transcription by binding to the promoter of the death associated protein kinase (DAPK) gene, a tumor suppressor that is silenced in many cancers (Kotarba et al., 2018). Besides, TFCP2 can positively regulate the transcription of p21^CIP1^, a well-known cell cycle inhibitor protein (Goto et al., 2016; Kotarba et al., 2018). In addition, previous studies have shown that Klotho can inhibit the IGF-1/insulin pathway and regulate the expression of Bax/Bcl-2, thereby inhibiting cell proliferation and promoting apoptosis in A549 cells (Chen et al., 2010). While recently Wang et al. (Wang et al., 2021) found that circ-ITCH boosted Klotho expression though acting as a miR-199a-5p sponge, thus, suggesting that the function of circ-ITCH in GC may involve cell cycle-related regulatory proteins. In short, recent studies have discovered that circ-ITCH regulates the expression level of a number of cell cycle-related proteins, as a ceRNA, to induce tumor cell apoptosis and limit tumor cell growth, thereby acting as an anti-tumor agent.

### 2.4 Regulating Epithelial Mesenchymal Transition Process

Epithelial mesenchymal transition (EMT) is a process that occurs in almost all forms of tumors and is linked to tumor incidence, invasion, metastasis, recurrence, and medication resistance (Iwatsuki et al., 2010). E-cadherin and vimentin are two crucial proteins that are frequently used as EMT indicators (Iwatsuki et al., 2010). E-cadherin, which is encoded by CDH1, is involved in EMT and is linked to tumor invasion and diffusion (Ye et al., 2012). Vimentin, in particular, promotes EMT, whereas E-cadherin opposes it. Lin et al. (2020) discovered that circ-ITCH inhibits EMT in OC by increasing CDH1 expression via sponging miR-106a. Guo et al. (2022) revealed that the tumor suppressor role of circ-ITCH in HCC is associated with regulating EMT progression through KEGG enrichment analysis, and its regulation function is associated with sponging miR-184. It is well known that Klotho-mediated regulation of cellular EMT is a way to regulate tumor progression (Chen et al., 2019). Therefore, by modulating the miR-199a-5p/Klotho axis, circ-ITCH can block EMT and delay tumor growth in GC (Wang et al., 2021). In addition, earlier research has demonstrated that the Wnt pathway is important for regulating EMT (Kumari et al., 2021). As a result, circ-ITCH's modulation of the Wnt pathway might have an impact on the downstream EMT process, but more research is needed to confirm this.

**FIGURE 2:**
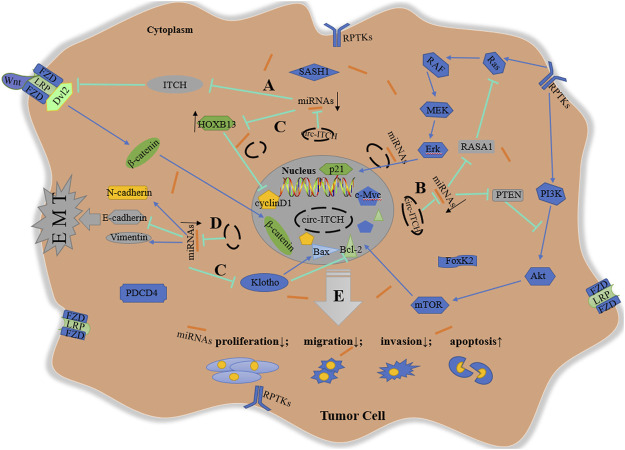
Schematic diagram of anti-tumor mechanism of circ-ITCH. **(A)** *via* sponging miR-7, miR-17, miR-20a, miR-22-3p, and miR-214, circ-ITCH increases ITCH levels to inhibit Wnt signaling pathway in ESCC, CRC, LC, TNBC, PCa, HCC and GC; **(B)** *via* sponging miR-7, miR-14, miR-22, miR-145 and miR-224, circ-ITCH activate the inhibitory proteins (RASA1 and PTEN) of Erk and PI3K cascade to suppress the activation of these signaling pathway in BCa, OS, NPC and OC; **(C)** *via* sponging miR-17-5p, miR-421, miR-524, miR-660, miR-106a-5p, miR-224-5p, miR-93-5p and miR-199a-5p, circ-ITCH regulates cell cycle-related proteins to inhibit cell proliferation and promote cell apoptosis in PCa, OS, OSCC, HCC, ccRCC, CC, glioma, melanoma and GC; **(D)** *Via* sponging miR-184 and miR-199a-5p, circ-ITCH suppresses EMT process in HCC and GC; **(E)** In addition, circ-ITCH can directly regulate some proteins without specific target miRNAs. In a word, circ-ITCH plays an anti-tumor role by negatively regulating cell proliferation, invasion, migration, and positively regulating cell apoptosis.

In summary, circ-ITCH modulates downstream targets including the Wnt pathway, the PI3K/Akt cascade, the MEK/Erk cascade, cell cycle-related proteins, and EMT process via sponging different miRNAs, performing an anti-tumor effect in a range of malignant tumors (Figure 2).

## 3 Regulation Mechanism and Role of Circ-ITCH in Non-Tumor Diseases

### 3.1 Bone Diseases

Osteoporosis is a systemic bone disease that causes decreased bone density and quality, disturbed bone microarchitecture, and increased bone fragility, all of which increase the risk of fracture (Compston et al., 2019). Zhong et al. (2021) demonstrated that compared to normal tissues circ-ITCH expression was down-regulated in osteoporosis samples, implying that it may play a protective role in bone degenerative diseases. Specifically, circ-ITCH up-regulated the expression of YAP1 by sponging miR-214. YAP1 is a prominent downstream effector of the Hippo pathway, and its up-regulation can stimulate the differentiation of mesenchymal stem cells into osteoblasts, according to previous research (Lorthongpanich et al., 2019). Moreover, YAP1 stimulates osteogenesis though interacting with β-catenin in osteoblasts (Pan et al., 2018). Taken together, the study found that circ-ITCH might enhance osteogenic differentiation in osteoporosis and ameliorate osteoporosis symptoms in mice (Zhong et al., 2021). Similarly, circ-ITCH expression is up-regulated during periodontal ligament stem cell (PDLSC) osteogenic differentiation and may trigger osteogenic differentiation though regulating MAPK pathway (Gu et al., 2017).

Intervertebral disc degeneration (IDD) is a type of degeneration that can cause a variety of minor or self-limiting symptoms. Spinal discomfort is currently thought to be mostly caused by IDD. Degradation of the extracellular matrix (ECM) and apoptosis of the nucleus pulposus (NP) cells are other key markers of IDD development (Wang et al., 2020). Recently, Zhang et al. (2021) discovered that circ-ITCH might sponge miR-17-5p/SOX4 signaling to positively regulate the activation of Wnt/β-catenin pathway in IDD, causing ECM degradation and NP cell apoptosis. However, this finding contradicts prior findings, particularly in tumor research, in that it activates Wnt/β-catenin pathway. After Wnt/β-catenin activation, Zhang et al. were unable to further illustrate the regulatory mechanism. Combined with earlier research (Zimmerman et al., 2013), we speculate that activating Wnt/β-catenin promotes apoptosis by up-regulating the expression level of pro-apoptotic proteins BIM and Bax while down-regulating the expression level of anti-apoptotic proteins Mcl-1 and Bcl-xl.

### 3.2 Cardiac Diseases

CircRNAs have been linked to the development of a number of cardiac diseases, including atherosclerosis, myocardial damage, heart failure, and drug-induced cardiotoxicity, according to research (Min et al., 2021). The current emphasis of circ-ITCH research in cardiac diseases is ischaemia-reperfusion (I/R) injury and doxorubicin-induced cardiotoxicity (DOXIC). In I/R damage, a substantial amount of H2O2 can be created, aggravating oxidative stress injury (Wu et al., 2018). H2O2 caused apoptosis in H9c2 rat cardiac cells and reduced viability, ATP levels, and circ-ITCH expression in a recent study (Zhang and Wang, 2020). Furthermore, H2O2 treatment boosted the expression of Wnt3a, Wnt5a, and β-catenin (Zhang and Wang, 2020). Conversely, in H2O2 pretreatment H9c2 cells, overexpression of circ-ITCH reduced apoptosis and Wnt/β-catenin expression, suggesting that its cardioprotective effect is linked to the inactivation of Wnt/β-catenin signaling pathway in I/R injury (Zhang and Wang, 2020). Specifically, circ-ITCH reduced cardiomyocyte apoptosis in I/R injury by sponging miR-17-5p and then inactivating the Wnt/β-catenin signaling pathway (Zhang and Wang, 2020). Doxorubicin is an effective chemotherapeutic agent, but doxorubicin-treated patients are prone to cardiac toxicity and subsequently develop congestive heart failure (Yeh and Bickford, 2009). The two primary pathogenic processes leading to the pathogenesis of doxycycline have been identified in DOXIC as oxidative stress and DNA damage (Zhang et al., 2012). Recently, Han et al. (2020) discovered that overexpressed circ-ITCH reduces doxorubicin-induced oxidative stress and DNA damage in cells and mitochondria. They also discovered that via sponging miR-330-5p in DOXIC, circ-ITCH upregulated sirtuin 6 (SIRT6), Survivin, and sarcoplasmic/endoplasmic reticulum Ca2+-ATPase 2a (SERCA2a) (Han et al., 2020). SIRT6 has been found to reduce oxidative stress by activating Nrf2 and SOD2 proteins, two important endogenous anti-oxidant defense molecules (Rezazadeh et al., 2019; Tian et al., 2019). In addition, SIRT6 could also ameliorate DNA damage via activating PARP1, a key DNA repair enzyme (Tian et al., 2019). Reportedly, Survivin could inhibit doxorubicin-induced myocardial apoptosis and fibrosis (Lee et al., 2014). Additionally, SERCA2a could catalyze the hydrolysis of ATP and enhance cardiac contractility by binding to calcium translocating from the cytosol to the lumen of the sarcoplasmic reticulum (Reilly et al., 2001). Thus, circ-ITCH can alleviate DOXIC and has good potential as a therapeutic target in DOXIC.

### 3.3 Diabetic Microangiopathy

Diabetic patients’ long-term glucose management is suboptimal, which can lead to diabetic microvascular problems such as diabetic neuropathy, diabetic retinopathy (DR) and diabetic nephropathy (DN). One of the most prevalent microvascular consequences of diabetes, diabetic retinopathy (DR), is a chronic, progressive diabetes mellitus-induced leakage and occlusion of retinal micro-vessels, resulting in a series of fundus lesions. DR is a persistent microvascular inflammation and proliferative neovascularization of the retina (Semeraro et al., 2015). And there is an interaction between the two pathological process (Capitão and Soares, 2016). TNF-α has been shown to be an inflammatory factor that plays a major role in high-glucose environments, where it is linked to vascular inflammation, endothelial dysfunction, oxidative stress, and disruption of the blood-retinal barrier, and contributes to the progression of DR synergistically (Capitão and Soares, 2016). The major enzymes responsible for degrading the ECM, matrix metalloproteinase (MMPs), have been linked to inflammatory disorders and diabetes (Kowluru and Mishra, 2017). Among these MMPs, MMP-2 and MMP-9 were both significantly up-regulated in retinal cells under high glucose conditions (Giebel et al., 2005). By suppressing TNF-α, MMP-2 and MMP-9, Zhou et al. (Zhou L. et al., 2021) revealed that overexpression of circ-ITCH might prevent neovascularization and inflammation, hence delaying DR progression. DN refers to the microvascular consequences of diabetes mellitus, with microalbuminuria at its core, and renal impairment in a subgroup of patients. Long-term chronic inflammation has been linked to the advancement of DN in studies (Wada and Makino, 2013). Previous research has shown that overexpression of SIRT6 could stimulate M2 macrophage transformation, inhibit high glucose-induced mitochondrial dysfunction and cell apoptosis by activating AMPK, all of which help to reduce inflammation in DN (Fan et al., 2019; Ji et al., 2019). A recent study in diabetic mice produced with streptozotocin found that circ-ITCH reduced kidney inflammation and fibrosis through modulating the mir-33a-5p/SIRT6 axis (Liu et al., 2021). The role and mechanism of circ-ITCH in uncomplicated diabetes is also worth exploring, according to current research development.

### 3.4 Hirschsprung Disease

Hirschsprung's disease (HSCR) is caused by a lack of proliferation and migration of intestinal nerve cells (ENCC), which leads to the absence of peristalsis and colon defecation (Heanue and Pachnis, 2007). It then causes the proximal colon to expand and hypertrophy, eventually resulting in the formation of a megacolon. Rearranged during transfection (RET) has recently been identified as a major regulator of ENCC formation, and inactivating mutations in this gene could result in HSCR (Ohgami et al., 2021). Accumulating evidence indicates that circRNAs are dysregulated and play critical roles in the development of HSCR. Xia et al. (2022) revealed that circ-ITCH expression was dramatically reduced in HSCR tissues, and its overexpression greatly promoted the ability of proliferation and migration of 293T, SH-SY5Y cell lines. Mechanistically, circ-ITCH overexpression activated RET by sponging miR-146b-5p, thereby relieving HSCR progression (Xia et al., 2022).

All in all, in non-tumor diseases such as IDD, osteoporosis, I/R injury, DOXIC, DR, DN, HSCR, and others, circ-ITCH plays a more complex regulatory role. It can, for example, regulate distinct target proteins to cause different states of the same signal pathway. These findings imply that the expression level of circ-ITCH and its regulatory mechanism are disease-specific. The role and mechanism of circ-ITCH in non-tumor illnesses and PDLSC osteogenic development are shown in Table 2.

**TABLE 2:** Role and mechanism of circ-ITCH in non-tumor diseases and physiology.

Disease/Physiology	miRNA	Target Proteins/Signaling Pathway	Effect	Ref
Osteoporosis	miR-214	YAP1	Promoting osteogenic differentiation	Zhong et al. (2021)
IDD	miR-17-5p	SOX4; Wnt/β-catenin (activating)	Promoting ECM degradation and NP cell apoptosis	Zhang et al. (2021)
Myocardial I/R injury	miR-17-5p	Wnt/β-catenin (inactivating)	Enhancing cardiomyocyte viability and ATP concentration; Inhibiting cardiomyocyte apoptosis	Zhang et al. (2020)
DOXIC	miR-330-5p	SIRT6, Survivin, SERCA2a	Alleviating cell/mitochondrial oxidative stress and DNA damage	Han et al. (2020)
DR	--	MMP-2, MMP-9, TNF-α	Preventing neovascularization and inflammation to delay DR progression	Zhou et al. (2021a)
DN	miR-33a-5p	SIRT6	Ameliorating renal inflammation and fibrosis	Liu et al. (2021)
HSCR	miR-146b-5p	RET; MAPK	Promoting cell proliferation and migration to delay HSCR progression	Xia et al. (2022)
PDLSC	--	MAPK	Promoting osteogenic differentiation	Gu et al. (2017)

## 4 Clinical Application and Perspective

With the increasing popularity of RT-PCR, diagnosing a growing range of diseases has gotten easier. RT-PCR can also be used to determine the degree of circ-ITCH expression. It also shows that it is practical and convenient because it expresses consistently in sample tissues, peripheral blood, and exosomes from patients. As a result, RT-PCR can be employed in practice to detect the level of circ-ITCH expression in patients’ sample tissues, peripheral blood, or exocrine, allowing for early diagnosis. The clinical significance of circ-ITCH in human diseases is shown in Table 3.

**TABLE 3:** Clinicopathological features related to circ-ITCH in human diseases.

Tumor Types	TNM Stage			Clinical Stage (*p* value)	Tumor Grade (*p* value)	Overall survival (*p* value)	Disease-free Survival (*p* value)	AUC (*p* value)	Ref
	T (*p* value)	N (*p* value)	M (*p* value)						
OC	*p* = 0.0009			*p* = 0.0021		*p* = 0.0257			Lin et al. (2020)
BCa					*p* = 0.034				Yang et al. (2018)
TNBC	*p* = 0.016	*p* = 0.008		*p* = 0.002		*p* = 0.01			Wang et al. (2019a)
NSLC	*p* < 0.001	*p* < 0.001		*p* = 0.003		*p* = 0.006	*p* = 0.001		Li et al. (2019)
OSCC	*p* = 0.035			*p* = 0.027					Hao et al. (2020)
PCa	*p* = 0.002	*p* = 0.047				*p* < 0.001	*p* < 0.001	0.812	Huang et al. (2019)
EOC	*p* = 0.005			*p* < 0.001		*p* = 0.003			Luo et al. (2018a)
GC	*p* = 0.0216	*p* = 0.034			*p* = 0.02			0.7055 (tissues); 0.6538 (serum)	Ghasemi et al., 2019; Peng and Wang 2020; Wang et al. (2021)
HCC						*p* < 0.001			Guo et al. (2017)
MM						*p* = 0.018	*p* = 0.017	0.809	Zhou et al. (2020)
Hepatitis C virus infection	positively correlated with ALT, AST level (*p* < 0.001)							0.661	Sharkawi et al. (2022)

### 4.1 In Tumor Diseases

#### 4.1.1 Diagnostic Biomarkers

Although surgery, chemoradiotherapy, targeted therapy, and the therapeutic outcome of tumor patients have all improved over time, overall survival and quality of life remain a serious problem for patients. Hence, it is critical to diagnose and treat patients as soon as possible. In recent years, scientists have experimented with many approaches to improve the detection and surveillance of early malignant tumors, including radiation, immunology, and biomarkers. Diagnostic biomarkers have received a lot of attention among them. Many circRNAs, notably circ-ITCH, have shown tremendous promise as diagnostic biomarkers in various investigations (Tang et al., 2019).

Many studies have anticipated and proven the clinical application potential of circ-ITCH as a diagnostic biomarker due to its down-regulation in a variety of malignancies (Huang et al., 2019). In GC, circ-ITCH in tissue and serum-derived exosomes were explored separately for their diagnostic value. Among them, the AUC for detecting circ-ITCH in tissues was 0.7055 (sensitivity: 52.71%, specificity: 74.55%); the AUC in serum-derived exosomes was 0.6538 (sensitivity: 42.42%, specificity: 90.91%) (Wang et al., 2021). In multiple myeloma (MM), the AUC was 0.809 (sensitivity: 59.8%, specificity: 80.0%) (Zhou et al., 2020). In PCa, circ-ITCH showed higher diagnostic value (AUC = 0.812 (95% CI: 0.780–0.845)), and its low expression was linked to a higher probability of lymph node metastases (*p* = 0.047) and an advanced T stage (*p* = 0.002) (Huang et al., 2019). Furthermore, the expression of circ-ITCH has been linked to tumor size, tumor grade, TNM stage and clinical stage. Specifically, in OC, the expression of circ-ITCH was linked to tumor size (*p* = 0.0009) and clinical stage (*p* = 0.0021) (Lin et al., 2020); in TNBC, it was linked to tumor size (*p* = 0.016), lymphatic metastasis (*p* = 0.008) and clinical stage (*p* = 0.002) (Wang S. et al., 2019); in OSCC, it was correlated with clinical stage (*p* = 0.027) and lymphatic metastasis (*p* = 0.035) (Hao et al., 2020); in EOC, it was associated with tumor size (*p* = 0.005) and International Federation of Gynecology and Obstetrics (FIGO) stage (*p* < 0.001) (Luo et al., 2018a); in GC, it was correlated with tumor grade (*p* = 0.02), T stage (*p* = 0.0216) and lymphatic metastasis (*p* = 0.034) (Ghasemi et al., 2019; Peng and Wang, 2020; Wang et al., 2021); in NPC, it was correlated with lymphatic metastasis (*p* = 0.0021), clinical stage (*p* = 0.0028) and bone metastasis (*p* = 0.0285) (Wang et al., 2022); in non-small cell lung cancer (NSCL), it was linked to tumor size (*p* < 0.001), lymphatic metastasis (*p* < 0.001) and clinical stage (*p* = 0.003) (Li et al., 2019); in MM, it was correlated with international staging system (ISS) stage (*p* = 0.036) (Zhou et al., 2020). Guo et al. (2017), on the other hand, discovered that the single nucleotide polymorphisms rs10485505 and rs4911154 of circ-ITCH were strongly related with an elevated risk of HCC, suggesting that it might be employed as a biomarker for HCC susceptibility. Similarly, single nucleotide polymorphisms of rs4911154 of circ-ITCH could aggravate the malignant transformation from thyroid nodule (TN) to thyroid cancer (Guo et al., 2021). Furthermore, circ-ITCH's collaboration with established diagnostic indicators like carcinoembryonic antigen (CEA) and carbohydrate antigen 19–9 (CA19-9) may also boost diagnostic power. All in all, these findings indicate that it has a wide range of diagnostic utility in a variety of tumors, even in cancer susceptibility prediction, since it’s convenient and non-invasive.

#### 4.1.2 Prognostic Biomarkers

With the rapid growth of incidence rate and mortality rate of malignant tumors, its overall prognosis will be the main determinant of global public health and life expectancy. Surgery is the most effective treatment for malignant tumors, but recurrence and metastasis have a significant impact on the prognosis (Lasithiotakis et al., 2014). Recent studies have found that circ-ITCH is closely linked to clinicopathological characteristics and can be employed as a tumor prognostic biomarker, which will aid in tumor treatment. The expression of circ-ITCH is linked to the prognosis of a range of tumors, including HCC, EOC, PCa, BCa, OC, and so on, according to Kaplan-Meier survival analysis (Guo et al., 2017; Luo et al., 2018a; Yang et al., 2018; Wang S. et al., 2019; Wang X. et al., 2019; Huang et al., 2019; Lin et al., 2020). These findings imply that reduced circ-ITCH expression was associated with lower overall survival (OS) and disease-free survival (DFS). Specifically, decrease in circ-ITCH was associated with worse OS (*p* = 0.018) and DFS (*p* = 0.017) in MM patients (Zhou et al., 2020); in NSLC, its down-regulation was correlated with worse OS (*p* = 0.006) and DFS (*p* = 0.001) (Li et al., 2019); in PCa, its down-regulation was correlated with worse OS and DFS (both *p* < 0.001) (Huang et al., 2019); in EOC, its down-regulation was correlated with worse OS (*p* = 0.003) (Luo et al., 2018a). Furthermore, 1604 patients with various malignancies were included in a meta-analysis, which yielded the same results. Patients with reduced circ-ITCH expression had a lower OS (HR = 2.45, 95% CI: 2.07–2.90, *p* ≤ 0.01, univariate analysis; HR = 2.69, 95% CI: 1.82–3.96, *p* ≤ 0.01, multivariate analysis) (Sun et al., 2021). Taken together, these results suggest its promising value as a prognostic biomarker.

#### 4.1.3 Therapeutic Target

Many molecules and signal pathways may be appropriate for targeted therapy as our understanding of tumor development improves. Circ-ITCH is a promising therapeutic target since it has an anti-tumor impact that is connected to a range of substances and pathways, as evidenced by recent studies. Up-regulation of circ-ITCH to inhibit proliferation, invasion and migration of HCC cells, for instance, is one of the mechanisms by which lidocaine treats HCC (Zhao et al., 2021). Besides that, it also has great potential in improving chemoresistance and side effects of chemotherapy. Circ-ITCH can decrease MM cell growth and improve MM cell chemosensitivity to bortezomib (BTZ) (Liu et al., 2020). Furthermore, circ-ITCH can also alleviate the symptom of DOXIC, suggesting that it can be used simultaneously with chemotherapeutic drugs to alleviate chemotherapeutic side effects (Han et al., 2020). At present, noncoding RNA (ncRNA) therapy focuses primarily on alternative and inhibitory therapies (Ning et al., 2019). Circ-ITCH's alternative therapy is projected to play a significant role in tumor therapy since it suppresses tumor cell proliferation, increases apoptosis, and slows tumor growth by targeting a range of pathway molecules.

### 4.2 In Non-Tumor Diseases

According to recent research, circ-ITCH also has an important role in non-tumor tissue. These findings, together with circ-ITCH's stable expression in patient tissues and peripheral blood, as well as well-defined regulatory mechanisms, point to its potential as a biomarker. In Hepatitis C virus infection, for instance, circ-ITCH expression was positively correlated with liver enzymes AST, ALT (*p* < 0.001) and child grade. With AUC = 0.661 (sensitivity: 65 percent, specificity: 70 percent), circ-ITCH has diagnostic significance in plasma of Hepatitis C virus infection (Sharkawi et al., 2022). However, no studies have looked at the possibility of circ-ITCH as predictive biomarkers in non-tumor diseases, and this is currently a blank area of research. Circ-ITCH has been reported to play well-defined regulatory roles in bone illnesses, cardiac diseases, diabetic microangiopathy, and Hirschsprung disease, indicating that they could be therapeutically targeted. Specifically, circ-ITCH replacement therapy appears to have promise as a treatment for DOXIC. Moreover, further experiments with circ-ITCH replacement drugs are also worth studying.

## 5 Conclusion

The focus of this review is on how circ-ITCH, a circular RNA, regulates gene expression in the post-transcriptional stage by acting as a sponge for miRNAs, blocking them from binding to their target mRNAs. The role of circ-ITCH in cell proliferation, apoptosis, invasion, migration, and EMT regulation, as well as related signaling pathways, is then explored. Circ-ITCH's potential as a diagnostic and predictive biomarker in tumor and non-tumor diseases is then confirmed. Furthermore, circ-ITCH has a lot of potential in disease treatment because of its well-defined regulatory mechanism, notably in terms of enhancing chemosensitivity and reducing chemotherapy adverse effects in malignant tumor. These discoveries not only illuminate the molecular basis of circ-ITCH, but also pave the path for future clinical applications.

Based on the current research, we put forward some promising future research directions. To begin, the up-regulated biomarker is more ideal for clinical detection, but the down-regulated index can still be employed as long as the critical value is evident. As a result, it is crucial to explore the critical value of circ-ITCH in both patients and healthy people. Then, researchers should increase the sample size and AUC detection as much as possible in order to achieve a more accurate association between circ-ITCH and other classic diagnostic markers (CEA, CA19-9) or prognostic indicators (OS, DFS) (Li et al., 2020c). Furthermore, given its critical role in regulating human diseases, more simulations of activators or carrier administration are needed to validate their *in vitro* and *in vivo* effects. While circ-ITCH is low expressed in a number of malignancies, it is unclear which tumor has greater specificity and accuracy about circ-ITCH, meriting additional investigation. At the same time, it is also worthwhile to explore the translational applications of circ-ITCH in chemotherapy because of its demonstrated potential in tumor chemotherapy, including chemo-sensitization and alleviation of side effects. Besides, due to the increase of ITCH concentration and regulating epidermal keratinocyte differentiation, the potential of circ-ITCH in decreasing radiotherapy injury also needs to be developed. Finally, it was recently shown that circRNAs are plentiful and stable in exosomes, that they can be produced under a variety of physiological and pathological conditions, and that they can be detected in circulation and urine (He et al., 2021), all of which require further exploration.
